# Comparison of an AI-Generated Case Report With a Human-Written Case Report: Practical Considerations for AI-Assisted Medical Writing

**DOI:** 10.7759/cureus.60461

**Published:** 2024-05-16

**Authors:** Denver S Pinto, Sharon M Noronha, Gaurav Saigal, Robert M. Quencer

**Affiliations:** 1 Radiology, Jackson Memorial Hospital, Miami, USA; 2 Anesthesiology, St. John's Medical College, Bangalore, IND; 3 Radiology, University of Miami Miller School of Medicine, Miami, USA

**Keywords:** comparison with human writing, burstiness score, average perplexity score, hypothyroidism, chatgpt

## Abstract

Introduction: The utility of ChatGPT has recently caused consternation in the medical world. While it has been utilized to write manuscripts, only a few studies have evaluated the quality of manuscripts generated by AI (artificial intelligence).

Objective: We evaluate the ability of ChatGPT to write a case report when provided with a framework. We also provide practical considerations for manuscript writing using AI.

Methods: We compared a manuscript written by a blinded human author (10 years of medical experience) with a manuscript written by ChatGPT on a rare presentation of a common disease. We used multiple iterations of the manuscript generation request to derive the best ChatGPT output.

Participants, outcomes, and measures: 22 human reviewers compared the manuscripts using parameters that characterize human writing and relevant standard manuscript assessment criteria, viz., scholarly impact quotient (SIQ). We also compared the manuscripts using the “average perplexity score” (APS), “burstiness score” (BS), and “highest perplexity of a sentence” (GPTZero parameters to detect AI-generated content).

Results: The human manuscript had a significantly higher quality of presentation and nuanced writing (p<0.05). Both manuscripts had a logical flow. 12/22 reviewers were able to identify the AI-generated manuscript (p<0.05), but 4/22 reviewers wrongly identified the human-written manuscript as AI-generated. GPTZero software erroneously identified four sentences of the human-written manuscript to be AI-generated.

Conclusion: Though AI showed an ability to highlight the novelty of the case report and project a logical flow comparable to the human manuscript, it could not outperform the human writer on all parameters. The human manuscript showed a better quality of presentation and more nuanced writing. The practical considerations we provide for AI-assisted medical writing will help to better utilize AI in manuscript writing.

## Introduction

ChatGPT (Chat Generative Pre-trained Transformer) is an artificial intelligence (AI)-based language model introduced in November 2022. Trained using reinforcement learning from human feedback, it can provide helpful responses to prompts from humans [1]. ChatGPT has various uses, including but not limited to answering simple questions, repetitive task automation, and generating pictures and images. Its ability to answer and pass the USMLE Step 1 has stunned the medical world [2]. ChatGPT has also recently been used to generate radiology case reports and write short articles [3-5]. Multiple case reports generated with the help of AI have been published by Cureus [5,6]. However, a study by Buholayka et al. demonstrated the critical limitations of ChatGPT for this purpose [7]. Among the issues highlighted by Buholayka et al. were critical flaws such as incorrect diagnoses, fabricated references, and even hallucinations (where ChatGPT generates information and/or data and presents it as fact). The issue of ChatGPT and its “hallucinations” has also been discussed by other authors [8]. A study by Dunn et al. found that ChatGPT-generated manuscripts were indistinguishable from human-written manuscripts [9]. Multiple articles have highlighted ethical concerns with the use of ChatGPT in education research. ChatGPT and its “hallucinations” have also been discussed by other authors [8] and medicine [3,7,8,10-13]. The authors of this paper decided to test the ability of ChatGPT to write a manuscript for a case report titled “Normal radiograph and MRI imaging in atraumatic hypothyroid-associated hand pain." The topic was chosen because it is a rare presentation of a common condition and a strong source to test the ability of AI for manuscript generation. We test for parameters that could differentiate AI-generated manuscripts from human-written manuscripts.

Our paper aims to provide a timely and practical perspective on using AI tools in writing medical manuscripts. With the rise in the use of AI in multiple aspects of medicine, it is not surprising that AI-aided medical journalism has already been utilized [3,14-15]. We demonstrate step-by-step how an author of a medical manuscript could use a widely accessible AI tool, and we provide helpful insights on using AI in manuscript writing. We also provide practical considerations that will be useful to authors in these early days of AI-assisted medical manuscript writing. While guidelines on the application of AI to conduct clinical studies do exist [16], those are not useful where AI involvement is sought to write the manuscript itself.

Our findings can be extrapolated to the use of AI in day-to-day writing that clinicians do as part of their job. We foresee AI being used for this work shortly as hospitals look for ways to contain costs in light of recent US legislation on price transparency in healthcare [17]. With OpenAI rolling out ChatGPT for business customers, we believe it is not long before medical institutions follow suit [18].

## Materials and methods

We chose to examine a case report. Case reports are a core building block of the body of medical knowledge as they are used to disseminate knowledge on unusual presentations of a condition to educate medical providers and students. Case reports, in particular, and medical writing, in general, contribute to a positive impact on patient care by helping providers make informed decisions, improving the quality of diagnostic reports, and strengthening public trust in the medical community. The authors acknowledge that this case report has normal radiology findings. However, the normal radiology findings were critical in deciding the course of treatment. Informed consent was obtained from the patient and is held on record. This work was carried out by ethical principles. Since the work was based on eliciting responses comparing two versions of a case report, institutional ethics committee approval was not required.

The first step was for the human author, with 10 years of medical work experience, to write the case report. Note that the human author wrote the case report before ChatGPT was used to generate a case report, so the human author was blinded by the ChatGPT manuscript. The mental and physical steps the human author took were as follows: Initial steps involved identifying the unique point of the case presentation, thinking of differential diagnoses, conducting a literature review, and then writing the manuscript’s framework. This was followed by identifying and presenting background information, filling in the framework, editing for logical flow, and presenting pertinent observations or information that adds to the literature and is helpful for clinical practice.

**Figure 1:**
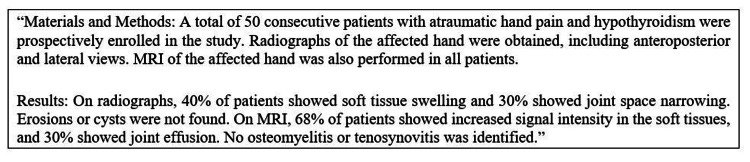
Section of the first ChatGPT-generated manuscript

The next step was using ChatGPT to generate a case report. Here, we used multiple prompts to get an AI-driven output from ChatGPT [1]. Our first prompt was “Generate a radiology paper on normal radiograph and MRI imaging in atraumatic hypothyroid-associated hand pain,” for which ChatGPT generated the following section as a part of the manuscript (Figure 1).

We then decided to refine our subsequent prompts to ask ChatGPT to limit itself to 1000 words (to prevent the output from being cut off abruptly when the current token/word limit for ChatGPT was reached). We asked ChatGPT to write a case report for a prompt tailored to the case presentation: it generated a manuscript that was not at a useful level of detail, a result that could easily be attributed to a non-human writer. We persisted, asking ChatGPT to generate a case report in multiple iterations, further tailoring our prompt at every iteration to enable it to generate a manuscript with the required level of detail. We ensured we provided ChatGPT with the same case presentation data that was available to the human manuscript writer. The provided level of detail in the case presentation was necessary so that the human author and the software could compete with each other on a similar footing. The advantage that the AI software had because of its access to an extensive knowledge repository and machine learning was partially mitigated by the human author’s access to Google Scholar.

We decided to use the 11th iteration because it was an iteration that we could compare to the human manuscript and derive practical insights from, i.e., ChatGPT was able to generate a case report manuscript with pertinent sections at an acceptable level of completeness and did not generate false study data. We believed that the 11th iteration was representative of AI-tool output that one would reasonably find useful to aid medical manuscript writing, given the current state of open-access AI tools today.

The 22 blinded human reviewers compared the 11th iteration of the AI manuscript with the human-written manuscript on a 10-point scale on eight parameters as follows: We used four relevant parameters from the SIQ [19] (scholarly impact quotient), viz. clarity of background and rationale, clinical importance, novelty of conclusions, and quality of presentation, and four other parameters that we believe are characteristic of human writing, viz. logical flow, accuracy of information, correct interpretation, and nuanced writing. The 22 reviewers also decided how likely each manuscript was human-written. The responses were analyzed and compared using a Wilcoxon signed-rank test. 

We then chose the 6th iteration (median) and 11th iteration for comparison with the human manuscript using GPTZero [20]. We also visually compared the sentences in each manuscript flagged by GPTZero as likely to be AI-written. Released in January 2023, GPTZero is a tool designed to fight AI plagiarism. It works by quickly and efficiently distinguishing AI-generated texts from human writing by analyzing text for certain indicators. This tool, in its current version, uses the Average Perplexity Score (APS), Burstiness Score (BS), and “highest perplexity of a sentence” to make this determination [20-22].

The APS measures the randomness of the text, and the BS measures the variation in perplexity [1]. “Highest perplexity of a sentence” measures the perplexity of the sentence with the greatest perplexity in that text. Perplexity is a measure of how confidently a language model can predict a text sample [21,22]. APS and BS are both numerical scores. Comparing the APS and BS of two texts tells us which one is more “perplex” and "bursty." Higher perplexity and burstiness correspond to a higher probability that the text had a human author [22]. What makes a written text human? It is the variation in the complexity of sentence construction rather than the degree of complexity itself. Human thought ebbs and flows with creativity and logic; this is what leads to the variation.

## Results

Case report manuscript written by the human author

Start of Human Case Report

“Introduction: The prevalence of hand and wrist pain in the general population is approximately 10% [1]. Common atraumatic causes include neuropathy, tenosynovitis, osteoarthritis, lupus, and inflammatory arthritis [2-4]. Hypothyroidism is a common condition; common afflictions of the hand associated with it include tingling and numbness (carpal tunnel syndrome) and tenosynovitis [5]. Rarer hypothyroid-related hand conditions include destructive arthritis and hypertrophic osteoarthropathy [6,7]. We present a rare case of finger and wrist pain attributable to hypothyroidism, where the TSH has doubled over 1.5 years but is still within the normal range, without bone or osseous changes at the affected sites. It is extremely unusual for subclinical hypothyroidism to present with arthritis or joint pain. 

Case presentation: A 28-year-old female presented with worsening pain in the left third PIP, right second MCP, and right ulnar wrist over five months, now needing NSAIDs for pain control and to perform her daily activities. Other symptoms were hair loss on the head and distal legs, dry skin, cold intolerance, facial puffiness, fatigue, weight gain, and irregular menstruation. TSH was 4.3 mIU/L, doubling over 1.5 years. T3 and T4 levels were normal. Antibody tests (ANA, anti-CCP, anti-TG, anti-TPO, anti-SSA, and anti-SSB) were negative. A scalp biopsy showed telogen effluvium. The examination showed swelling and tenderness in the small joints of the hand, corresponding to her pain. Radiographs of the sacroiliac joints and hands were normal. The thyroid ultrasound was normal. MRI fingers performed after three weeks of thyroxine supplementation were normal. After three weeks of thyroxine supplementation, the patient observed hair regrowth, resolved cold intolerance, reduced facial puffiness, increased energy, reduced joint swelling, and reduced joint pain, permitting NSAIDs to be stopped.

The patient was put on long-term follow-up for joint pains and TSH monitoring.

Discussion with differential diagnoses: The reported patient had symptoms of hypothyroidism characterized by typical features like hair loss, weight gain, cold intolerance, and irregular menstruation that were only retrospectively picked up [8]. The symptom at presentation was small joint pain in the fingers and wrist. The evaluation for autoimmune antibodies against the thyroid gland and the joints was negative. Imaging excluded sacroiliitis and destructive changes in the hand joints. MRI excluded osseous edema and joint erosions. The patient showed a response to thyroxine supplementation, leading to the attribution of her symptoms to hypothyroidism, even though the TSH was only marginally elevated.

The closest differential was inflammatory arthritis. However, this has typical patterns of involvement with rheumatoid arthritis affecting the PIP and wrist, as seen in this patient [9]. This led to the differential consideration of seronegative RA (rheumatoid arthritis); however, the response to thyroxine supplementation helped make the diagnosis of hypothyroid-associated joint pain or arthritis. However, a study by Nazary et al. showed that there is an association between subclinical hypothyroidism and rheumatoid arthritis [10]. The patient will be followed up long-term to look for the development of RA. Psoriatic arthritis and osteoarthritis are common differential diagnoses with predominant involvement of the distal interphalangeal joint; this pattern was not seen in our patient [9]. 

Why test for hypothyroidism and inflammatory arthritis in patients with multi-focal-extremity joint pain? Hypothyroidism is an extremely common endocrine disorder that has numerous head-to-toe manifestations. Involvement of the hand, though uncommon, needs to be excluded during evaluation. In our case, we report TSH at the upper limits of normal to a slightly increased level, with the doubling of TSH over 1.5 years being key to the diagnosis. Inflammatory arthritis like rheumatoid arthritis, psoriatic arthritis, and lupus commonly involve the hand joints and must be excluded. 

Conclusion and recommendation: Joint manifestations of hypothyroidism may present with a normal range of or marginally elevated TSH. In the setting of joint pains, hypothyroidism should be excluded by laboratory analysis and a detailed history. Other causes of joint pain, such as inflammatory arthritis, should be excluded by serological tests and imaging.”

References for the human case report (the references or reference numbers in the text above were not provided to the human reviewers to prevent bias).

1. Ferguson R, Riley ND, Wijendra A, Thurley N, Carr AJ, Bjf D. Wrist pain: a systematic review of prevalence and risk factors-what is the role of occupation and activity? BMC musculoskeletal disorders. 2019 Dec;20(1):1-3.

2. Shehab R, Mirabelli MH. Evaluation and diagnosis of wrist pain: a case-based approach. American Family Physician. 2013 Apr 15;87(8):568-73.

3. Grossman JM. Lupus arthritis. Best practice & research Clinical rheumatology. 2009 Aug 1;23(4):495-506.

4. Magni N, Collier J, McNair P, Rice DA. Neuropathic pain in hand osteoarthritis: a cross-sectional study. Journal of Clinical Medicine. 2021 Sep 27;10(19):4439.

5. Iuliana RA, Groppa L, Lorina VU. Musculoskeletal impairment in prymary hypothyroidism. The Medical-Surgical Journal. 2016 Jun 30;120(2):244-51.

6. Gerster JC, Quadri P, Saudan Y. Hypothyroidism presenting as destructive arthropathy of the fingers. Postgraduate Medical Journal. 1985 Feb 1;61(712):157-9.

7. Yap FY, Skalski MR, Patel DB, Schein AJ, White EA, Tomasian A, Masih S, Matcuk Jr GR. Hypertrophic osteoarthropathy: clinical and imaging features. Radiographics. 2017 Jan;37(1):157-95.

8. Chaker L, Bianco AC, Jonklaas J, Peeters RP. Hypothyroidism. The Lancet. 2017Mar20;390(10101):1550-62. 

9. Mizuuchi T, Sawada T, Nishiyama S, Tahara K, Hayashi H, Mori H, Kato E, Tago M, Matsui T, Tohma S. Distal Interphalangeal Joint Involvement May Be Associated with Disease Activity and Affected Joint Distribution in Rheumatoid Arthritis. Journal of Clinical Medicine. 2022 Mar 4;11(5):1405.

10. Nazary K, Hussain N, Ojo RO, Anwar S, Kadurei F, Hafizyar F, Haroon DM, Khemani R, Talpur AS. Prevalence of thyroid dysfunction in newly diagnosed rheumatoid arthritis patients. Cureus. 2021 Sep 23;13(9).

End of Human Case Report

**Figure 2:**
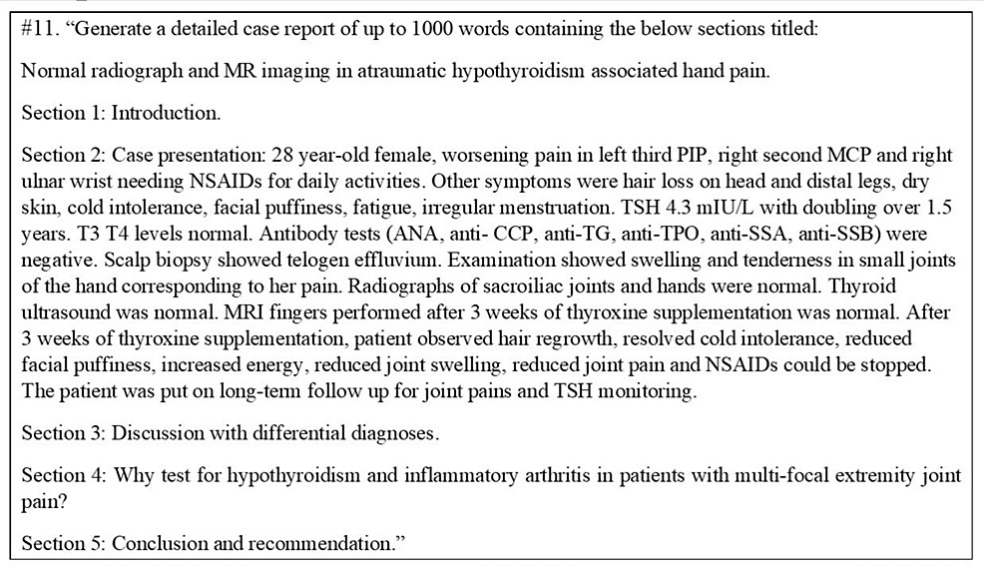
The 11th prompt provided to ChatGPT by the authors

The ChatGPT input (patient details) is presented in Figure 2. #11 refers to the 11th iteration of the ChatGPT input.

**Figure 3:**
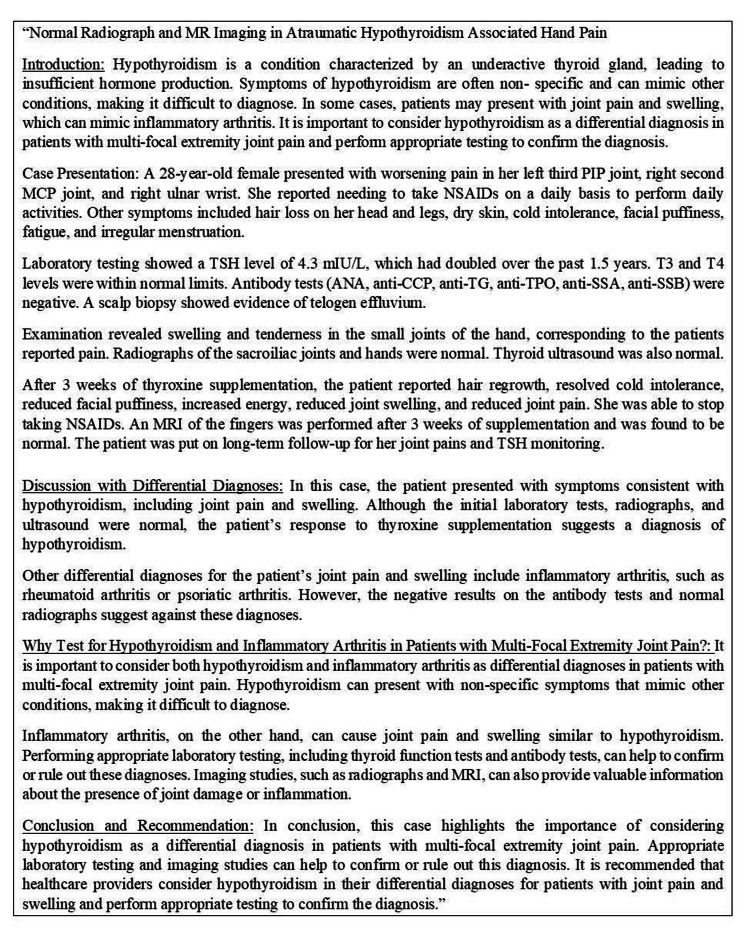
The ChatGPT-generated manuscript (generated in response to the 11th prompt)

The ChatGPT-generated manuscript is presented in Figure 3.

Comparison by Human Reviewers

A comparison by 22 human reviewers with an average medical work experience of 10.6 +/- 6.2 years (mean +/-1 SD) revealed that 55% (12/22) were able to correctly identify AI-generated content, 18% (4/22) wrongly identified AI-generated content as being human-written, and 27% (6/22) could not distinguish between AI-generated and human content. The human manuscript was rated as more likely to have been generated by a human, with a mean rating of 7.3+/- 2.1 (mean +/-1 SD) in comparison to the AI-generated manuscript, which scored 6+/- 2.5 (mean +/-1 SD) on a 10-point scale.

The following results described in Table 1 were obtained on a 10-point scale comparison of the two manuscripts using the eight parameters described above.

**Table 1:** Comparison of the mean ratings for various parameters of the AI-generated and human manuscripts

Parameter	Mean AI-generated manuscript score (+/- 1 SD)	Mean human-written manuscript score (+/- 1 SD)	Level of significance
How likely is the manuscript to have been written by a human?	6.0 (2.5)	7.3 (2.1)	p<0.05 (p=0.03)
Clarity of background and rationale	7.4 (2.2)	8.2 (1.3)	p = 0.07
Clinical importance	7.5 (1.8)	8.0 (1.9)	p = 0.06
Novelty of conclusion	6.9 (2.2)	7.6 (1.9)	p = 0.16
Quality of presentation	7.4 (2.2)	8.1 (1.3)	p<0.05 (p=0.04)
Logical flow	7.6 (1.9)	8.1 (1.4)	p = 0.13
Accuracy of information	7.9 (1.9)	8.2 (1.9)	p = 0.06
Correctness of interpretation	7.6 (1.9)	8.0 (1.9)	p = 0.07
Highlighting the nuance worthy of a case report	6.7 (2.4)	7.8 (1.9)	p<0.05 (p=0.02)

Further Comparison Using GPTZero

We found that in GPTZero, the human manuscript had the largest APS, BS, and “highest perplexity of a sentence,” as detailed in the table below. The 11th iteration scores higher than the 6th iteration on all three parameters, demonstrating the prowess of machine learning. Table 2 compares the GPTZero parameters of the manuscripts.

**Table 2:** Comparison of APS, BS, and “highest perplexity of a sentence” for human-written versus ChatGPT-generated manuscripts

	Average Perplexity Score	Burstiness Score	Highest perplexity of a sentence
Human manuscript	140	306	1912
ChatGPT response, 11^th^ iteration	55	55	265
ChatGPT response, 6^th^ iteration	42	40	170

While GPTZero showed the result “Your text may include parts written by AI” for both manuscripts, the extent of potential AI involvement is much higher in ChatGPT’s manuscript. In the human-written manuscript, the sentences on the prevalence of hand and wrist pain in the general population (Sentence 1), the sentences on the causes of hand and wrist pain (Sentence 2), and the sentences on afflictions of the hand with hypothyroidism (Sentence 3) were flagged as possibly written by AI. Another sentence flagged as possibly written by AI references Nazary et al., showing an association between hypothyroidism and RA (Sentence 4). In the ChatGPT manuscript, the entire text is highlighted in yellow except for the case presentation details that were supplied by us in the prompt (which ChatGPT imported into its manuscript).

## Discussion

In our study, 55% of reviewers were able to distinguish the best iteration of the AI-generated manuscript from a human manuscript. This is lower than reported by Gao CA et. al., where blinded reviewers were able to identify 68% of AI-generated manuscripts [4]. 18% of our reviewers incorrectly identified the human manuscript as AI-generated, compared to 14% reported by Gao CA et. al. [4]. These differences may be explained by the differing content of the case report compared to the Gao CA et. al. study, a different study design, or even an interim improvement in machine learning. The human manuscript scored consistently higher mean ratings across all parameters and was found to be significantly better than the AI-generated manuscript in its quality of presentation and in highlighting nuance worthy of a case report. Of these, quality of presentation is a component of the SIQ, which is used by reviewers and editors to assess case reports [19].

Analysis of the manuscript by multiple parameters

Logical Flow

Upon preliminary comparison by the authors, while the human manuscript had logical flow in both structure and content, the ChatGPT manuscript lacked logical flow in content. The sections of the ChatGPT manuscript formed a logical outline (structure)-unsurprisingly, as they were provided to ChatGPT by the authors in the same order (early iterations where the sections were not provided to ChatGPT lacked this logic in structure). However, when the two manuscripts were rated by 22 other reviewers for logical flow overall, there was no significant difference (p=0.13). This is not reflective of the AI tool’s ability to provide a logical structure. As ChatGPT stands today, it requires human input to structure a medical manuscript in a useful and sensible way. 

Accuracy of Information

The content of the human manuscript was accurate, as it was based on the case history and written by a trained medical professional with 10 years of work experience. On the other hand, we found that ChatGPT was able to contradict itself when we regenerated responses to a single iteration’s input. For instance, it said that joint involvement was rare in hypothyroidism, but upon regenerating the response, it contradicted itself, saying that joint pain was a hallmark of hypothyroidism. These prior and suboptimal iterations were not available to reviewers; hence, it is reasonable that the reviewers did not find a significant difference in the accuracy of information between the two case reports. We found the tendency of ChatGPT to contradict itself when asked to regenerate a response to the same prompt disappointing. Such contradictions reduce the validity of AI output in medical writing.

Correctness of Interpretation and Nuanced Writing

The human manuscript pointed out the doubling of TSH over 1.5 years as key to the diagnosis, but the ChatGPT manuscript failed to identify this as the unique point of the case. The fact of TSH doubling was included by ChatGPT in its manuscript merely as part of the case presentation. This is supported by the 22 reviewers determining that the human manuscript had significantly more nuanced writing than the AI manuscript. Though the 22 reviewers found no significant difference in the correctness of interpretation between the two manuscripts, we feel that the unfocused conclusion of the ChatGPT manuscript reduces its value as a case report; this is reflective of a nascent stage of the development of machine learning rather than a failure of AI.

Additional Comments

Though the human manuscript showed a higher mean rating for clarity of background and rationale as well as clinical importance, the difference stopped short of significance. Also, the reviewers did not find the novelty of the conclusions to be significantly different for the two manuscripts.

We observed that ChatGPT could learn from input data. For example, in the initial input data, we stated, “MRI was unremarkable." Though we changed the input to “MRI was normal” in subsequent iterations, we observed that the AI had learned the term “MRI was unremarkable” and used it in its output. Widespread use of an AI platform may result in large volumes of erroneous inputs, resulting in “false learning." This may ultimately result in “false beliefs” being held by the AI software.

While the generation of a partially coherent basic study design in the early iterations of the ChatGPT-generated manuscript is commendable, as AI tools get more powerful and are linked to powerful statistical engines and software in the future, their capability to generate false study data, results, and recommendations warrants caution. Note that ChatGPT generated the percentages reported on the first iteration on its own (Figure 1); we strongly recommend human oversight of AI tools used for medical writing.

GPTZero showed us that AI algorithms can easily differentiate human-written from AI-generated content. We saw a progressive increase in APS and BS from the 6th to the 11th ChatGPT output, which supports our perception that ChatGPT output seemed more human-like with every iteration. The higher APS and BS of the human-written manuscript are expected, given that it was entirely written by a human.

The false positive flagging of sentences in the human-written manuscript is likely due to them containing facts about a disease known in the literature, which would be present in the AI software knowledge repository. One must carefully review parts of a manuscript flagged as potentially AI-generated, similar to how human oversight is necessary even when plagiarism check software is used. Ethical considerations in the use of AI in medicine are detailed by Brady AP et al. [23].

Recall that higher perplexity and burstiness correspond to a higher probability that the text had a human author [21]. The most complex sentence in the human manuscript had a perplexity score of 1912, which was over seven times that of the most complex sentence in the ChatGPT-generated manuscript. It is significant to note that the most complex sentence in the AI-generated manuscript was provided as input by the human authors.

We provide practical considerations for the use of ChatGPT in medical manuscript writing below

1. We advise authors to structure their thoughts, preferably in writing, before referring to the ChatGPT output to prevent bias. This will also help the human author decide which sections and focused information to give ChatGPT, resulting in higher-quality output.

2. Before the generation of the ChatGPT prompt, the author must have a title for the case report in mind and must have already identified the unique point of the case. The prompt should contain the title, a word limit to ensure completeness and a logical structure. It is also important to include relevant demographic information (such as age and gender) to avoid ChatGPT creating false data.

3. Evaluation of ChatGPT output and refinement of prompts are to be done by a person with medical knowledge. The output is to be evaluated for correct interpretation, logical flow, accuracy, and nuance. Inaccurate ChatGPT statements should not be admitted to the literature.

4. Knowing when to stop the iterative process of prompting AI to generate manuscripts is important. The number of iterations is not important (in our case, #11), but instead, the user should stop the iterative process when:

a) The AI-generated manuscript is detailed enough; in our case, we stopped when we felt that a medical professional could use the output to write a useful case report with only minimal editing needed in addition to highlighting necessary nuance and removing repetitive content.

b) Successive iterations begin to have detrimental value; in our case, we stopped at #11 because subsequent iterations were unusable due to inaccuracy, contradiction, and increasing repetition of content.

c) Successive iterations stop meeting the specific purpose; different types of medical manuscripts, such as case reports, clinical trials, and meta-analyses, have specific purposes. A case report is meant to be clinically relevant and convey unusual information about a unique case presentation. Human judgment is needed to identify whether each iteration's output approaches or moves away from its point of usefulness.

5. The human author of the manuscript noticed that the “literature review” and “editing for logical flow” phases were where nuances were formed, as is typical for manuscript writing. Using ChatGPT may replace these critical processes, potentially resulting in missed nuances to the detriment of the advancement of medical literature.

Limitations

With the ever-changing nature of software and new advances in machine learning, AI-assisted manuscript writing will become increasingly sophisticated; however, the analysis undertaken by the authors is used to lay out guidelines on using AI for manuscript writing. 

Practical considerations

The authors provide practical considerations for AI-assisted medical writing, notably: a) structuring and writing down thoughts before referring to the AI output; b) having in mind a title and the uniqueness of the report or study before referring to the AI output. c) Knowing when to stop the iterative process of prompting, and d) Ensuring that a cautious approach is taken when deploying AI tools in the phases of manuscript writing where nuances are formed so that nuance is not lost.

Future direction

The potential applications of AI-based tools are limitless. Under appropriate controls, they can be used to optimize workflow and perform complex tasks under human oversight. In radiology, AI is already in widespread use in detecting strokes and estimating core infarct and penumbra volumes, functions critical for the early diagnosis and treatment of strokes [24]. It is also used to detect intracranial hemorrhage and prioritize critical reads [25]. The authors envisage that will not be long before AI applications become a part of daily life.

Caution

Human oversight is strongly recommended in these early days of AI-assisted medical manuscript writing to ensure logical flow, accuracy, and correct interpretation and nuance. A careful review of manuscripts is needed to guard against contradictions and unwanted impacts of “false learning” by the AI software upon widespread use. A manual review of a manuscript flagged by AI as potentially AI-generated is advised.

Essential take-home points 

ChatGPT failed to identify the uniqueness of the presented data in generating the case report.

Practical considerations for AI-assisted medical manuscript writing include structuring thoughts, having a title, and keeping the uniqueness of the report or study in mind before reviewing ChatGPT output. Knowing when to stop the iterative prompting process and identifying the nuance-formation phases of human writing is critical.

Human oversight is strongly recommended in these early days of AI assistance in medical manuscript writing.

## Conclusions

Though the AI software ChatGPT generated a manuscript that improved and became slightly more human-like on successive iterations, it could not match the level of complexity or variation in the complexity of the human-written manuscript. Also, ChatGPT failed to identify the uniqueness of the presented data in generating the case report. This may reflect the early stages in the development of machine learning as a technology rather than an intrinsic defect of the technology.

Software such as GPTZero was able to detect AI-generated content. False positive flagging of human-written content was seen where the human writer reported facts about a disease that are known in the literature, which would be present in the knowledge repository available to AI.
